# Rho-ROCK and Rac-PAK Signaling Pathways Have Opposing Effects on the Cell-to-Cell Spread of Marek's Disease Virus

**DOI:** 10.1371/journal.pone.0044072

**Published:** 2012-08-27

**Authors:** Nicolas Richerioux, Caroline Blondeau, Agnès Wiedemann, Sylvie Rémy, Jean-François Vautherot, Caroline Denesvre

**Affiliations:** 1 INRA, UMR1282, Infectious Diseases and Public Health, ISP, *BIOVA team,* Nouzilly, France; 2 INRA, UMR1282, Infectious Diseases and Public Health, ISP, *SPVB team,* Nouzilly, France; 3 Université de Tours, UMR1282, Infectious Diseases and Public Health, ISP, Tours, France; University of Bristol, Bristol, United Kingdom

## Abstract

Marek's Disease Virus (MDV) is an avian alpha-herpesvirus that only spreads from cell-to-cell in cell culture. While its cell-to-cell spread has been shown to be dependent on actin filament dynamics, the mechanisms regulating this spread remain largely unknown. Using a recombinant BAC20 virus expressing an EGFPVP22 tegument protein, we found that the actin cytoskeleton arrangements and cell-cell contacts differ in the center and periphery of MDV infection plaques, with cells in the latter areas showing stress fibers and rare cellular projections. Using specific inhibitors and activators, we determined that Rho-ROCK pathway, known to regulate stress fiber formation, and Rac-PAK, known to promote lamellipodia formation and destabilize stress fibers, had strong contrasting effects on MDV cell-to-cell spread in primary chicken embryo skin cells (CESCs). Inhibition of Rho and its ROCKs effectors led to reduced plaque sizes whereas inhibition of Rac or its group I-PAKs effectors had the adverse effect. Importantly, we observed that the shape of MDV plaques is related to the semi-ordered arrangement of the elongated cells, at the monolayer level in the vicinity of the plaques. Inhibition of Rho-ROCK signaling also resulted in a perturbation of the cell arrangement and a rounding of plaques. These opposing effects of Rho and Rac pathways in MDV cell-to-cell spread were validated for two parental MDV recombinant viruses with different *ex vivo* spread efficiencies. Finally, we demonstrated that Rho/Rac pathways have opposing effects on the accumulation of N-cadherin at cell-cell contact regions between CESCs, and defined these contacts as adherens junctions. Considering the importance of adherens junctions in HSV-1 cell-to-cell spread in some cell types, this result makes of adherens junctions maintenance one potential and attractive hypothesis to explain the Rho/Rac effects on MDV cell-to-cell spread. Our study provides the first evidence that MDV cell-to-cell spread is regulated by Rho/Rac signaling.

## Introduction

Marek's disease virus (MDV), also referred to as *Gallid herpesvirus 2*, is the causative agent of Marek's Disease (MD) in chickens, a multifaceted disease most widely recognized by the induction of a malignant T-cell lymphoma. MDV is a type-species of the *Mardivirus* genus (Marek's disease-like viruses) within the *Alphaherpesvirinae* subfamily of the *Herpesviridae* family. MDV replicates exclusively in avian cells, and efficiently to date only when they are explanted [1]. In these *in vitro* systems, MDV remains strictly cell associated without infectious enveloped particles being detectable in the supernatant and/or at the external cell surface [2], [3], [4]. Upon infection of the chicken host, MDV is likely transmitted in a cell-to-cell manner (e.g. between lymphocytes or from lymphocytes to skin), as no free virus can be detected in the plasma of infected birds [1]. MDV belongs to the rare category of viruses that does not spread in cell culture through the cell-free aqueous environment but only in the context of cell contacts between infected cells and naïve cells, a process termed “cell-to-cell spread”. While the detailed mechanisms of MDV cell-to-cell spread are still largely unknown, their elucidation would enhance our understanding of MDV biology.

To spread from cell-to-cell, viruses use either physiological cell-to-cell contacts and/or virus-driven contacts, that are permanent or transient (reviewed in [5], [6]). Filopodia, unique or multiple branched plasma membrane extensions, nanotubes, viral synapses and adherens junctions have been implicated in these processes [7], [8], [9], [10], [11], [12], [13]. For herpesviruses, depending on the *in vitro* infection system, various cell contact structures and routes have been described to be relevant for cell-to-cell spread. Both Pseudorabies Virus (PRV) and Herpes Simplex Virus 1 (HSV-1) were shown to induce long actin filaments-containing projections [8], [14], [15] and to traffic within it [8] or at their surface [15], respectively. Murine gamma-herpesvirus-68 (MHV-68) was shown to induce the outgrowth of long, branched, RhoA-dependent, actin-based plasma membrane fronds driven by the cytoplasmic tail of the viral gp48 glycoprotein [9], a protein which was also shown to promote viral spread [16]. For HSV-1, the virological synapse was shown to facilitate its entry into T-cells [7]. Finally, adherens junctions (AJs) were demonstrated to be a preferred route of HSV-1 dissemination because nascent HSV-1 virions are specifically sorted in this area in polarized HEC1A and MDBK epithelial cells [10]. In addition, the nectin-1, a component of AJs, was identified as a receptor for HSV-1 and HSV-2 glycoprotein D [17], [18]. All of these structures are dependent on filamentous actin (F-actin) for their formation and/or stability and some are directly modulated by one or several RhoGTPases signaling pathways.

F-actin is organized in three major structures in fibroblastic cells: stress fibers, lamellipodia, and filopodia [19]. All three structures are dynamic and their prevalence varies according to the cell type, physiology and environment [20]. The stress fibers/lamellipodia/filopodia balance is regulated by extracellular stimuli, including soluble factors and mechanical tensions, and involves the most studied RhoGTPases family members, Rho, Rac and Cdc42 (reviewed in [19], [21], [22]. In fibroblasts, RhoA activation promotes the formation of stress fibers, the maturation of focal complexes into focal adhesion and the maintenance of AJs [23], [24]; Rac1 activation yields to lamellipodia, dorsal ruffles formation, AJs initiation and formation of focal complexes [23], [25]. The activation of Cdc42 leads to the extension of filopodia [26]. RhoA, Rac1 and Cdc42, have several effectors, usually distinct, but sometimes common like p21-activated kinases (PAKs) for Rac1 and Cdc42, and mDia for Rho proteins and Cdc42 [27], [28]. The three Rho isoforms (A, B and C) have several common effectors as mDia and Rho-kinase (ROCK) 1 and 2 [29], which are both essential for stress fibers formation. mDia proteins catalyze the nucleation and polymerization of unbranched actin filaments for stress fibers and also for filopodia formation [30]. ROCK proteins promote and maintain stress fibers assembly as actomyosin contraction by phosphorylating both myosin light chain (MLC) and MLC phosphatase [31]. In contrast to ROCKs, activated PAK1 phosphorylates the myosin light chain kinase (MLCK), resulting in decreased MLCK activity and reduced actomyosin assembly [32].

For MDV, the contribution of actin filaments to viral cell-to-cell spread has thus far only been reported by Schumacher *et al.* [33]. The authors found that cytochalasin D (CytD) treatment, blocking actin polymerization, resulted in a reduction in the size and number of viral plaques. Given this result, our goal was to study whether actin organization and possibly actin dependent cell-cell contact, in relationship with RhoGTPases signaling, influence MDV cell-to-cell spread. For that, we used cells shown to be highly permissive for MDV replication, primary chicken embryo skin cells (CESCs) and measured MDV plaque size after modulating F-actin and Rho/Rac signaling pathways with different pharmacological agents. Our data show that MDV cell-to-cell spread is regulated in opposed manner by Rho/Rac signaling pathways and that efficient spread requires ROCKs signaling including myosin II activity.

## Results

### Actin filaments and their dynamics are important for MDV spread

**Figure 1:**
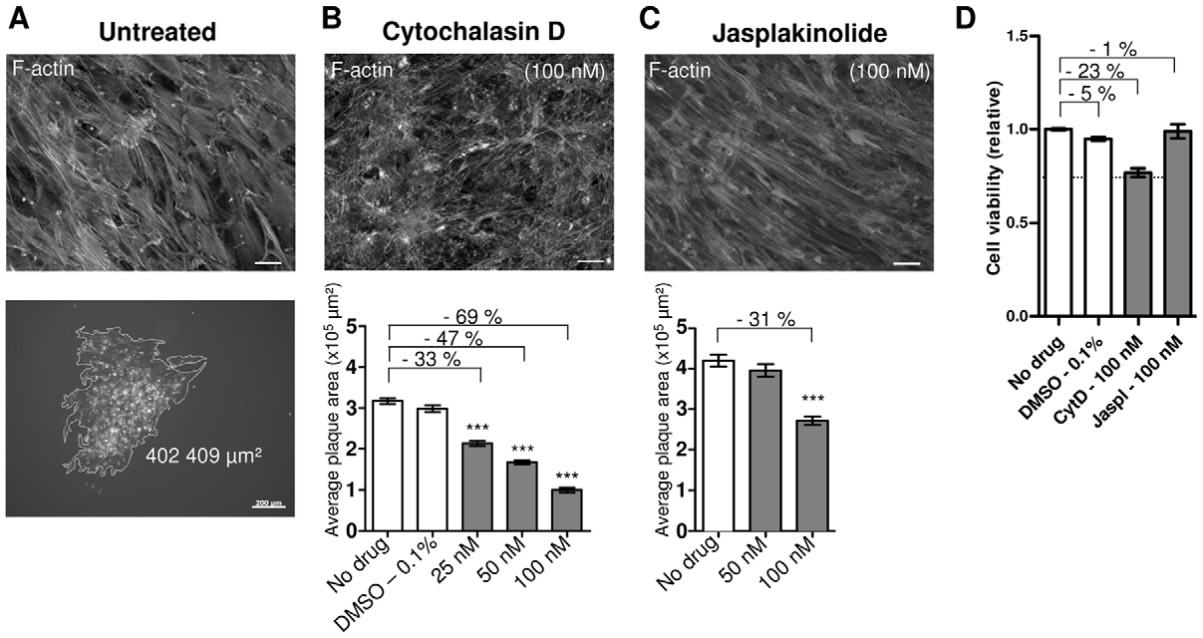
Modulators of actin polymerization decrease MDV cell-to-cell spread. CESCs monolayers were infected or not with the BAC20-EGFP for 6 hrs, and then treated for 4 days at the indicated drug concentration. (A) Upper panel, F-actin staining (AlexaFluor594-Phalloidin) in non-infected cells. Lower panel, representative example of a BAC20-EGFP plaque which area was measured in µm^2^. Bar, 200 µm. (B) Effect of CytD on actin polymerization and MDV cell-to-cell spread. Upper panel, F-actin staining of non-infected CESCs treated with 100 nM of CytD. Lower Panel, the graph shows a quantitative analysis of fifty plaques size in one of three representative experiments. Error bars represent the SEM. Percentages on graphs indicate the decrease (−) in plaques size compared to the untreated cells (***, p<0.001). (C) Effect of Jaspl. F-actin staining and plaques size are shown, as in (B). (D) The relative cell viability after a 4-day treatment with each drug at the highest concentration used. Values were normalized as compared to untreated cells. Error bars represent the SEM. Percentages on the graph indicate the decrease (−) in viability. The dashed line represents the acceptable CESCs viability limit (0.75) that was set in this study. For F-actin detection, bars, 20 µm.

Our MDV cell-to-cell spread model employs the co-culture of a CESCs confluent layer with GFP+-sorted infected cells at very low multiplicity of infection (moi) in order to get individualized plaques. The virus used was a BAC20-EGFP reporter expressing an EGFPVP22 protein [34]. MDV cell-to-cell spread was quantified by plaque size measurement, at 4 days post-infection (pi). Initially, we verified, using our MDV cell-to-cell spread assay, the effect on MDV spread of two agents that modulate F-actin formation. CytD is a fungal metabolite which prevents polymerization by reversibly binding to the barbed ends of F-actin and globular actin [35] while the jasplakinolide (Jaspl) stabilizes F-actin and induces actin polymerization [36]. We determined the most active and less-toxic concentration of these drugs on a dense monolayer of CESCs, monitoring the presence of actin filaments and the cell morphology. For each maximum drug concentration used, the cell viability was evaluated, after a 4-day treatment (Fig. 1).

The maximum tolerated concentration of CytD (100 nM) induced an incomplete disassembly of stress fibers (Fig. 1B), in comparison with untreated cells (Fig. 1A) and a 23% decrease in cell viability (Fig. 1D). Higher concentrations of CytD were incompatible with our spread assay because the CESCs rounded-up and detached from the plastic dish surface. After a 4-day treatment of CESCs with CytD, MDV dissemination was dose-dependently reduced. At 100 nM of CytD, MDV plaques were 69% smaller than those on untreated cells (Fig 1B), which is in good agreement with the previous report of Schumacher and collaborators [33]. The addition of Jaspl on CESCs preserved stress fibers and did not affect cell viability but resulted in the formation of cytoplasmic actin aggregates (Fig. 1C), as was previously reported for mammalian cells [37], [38]. Jaspl at 100 nM reduced MDV spread by 31% compared to untreated cells. Therefore CytD and Jaspl reduced MDV spread in our cell system and we confirmed that actin dynamic is important for MDV cell-to-cell spread.

### Actin cytoskeleton organization and cell-cell contacts are highly re-arranged in the center of MDV infection plaques but not in the periphery

**Figure 2:**
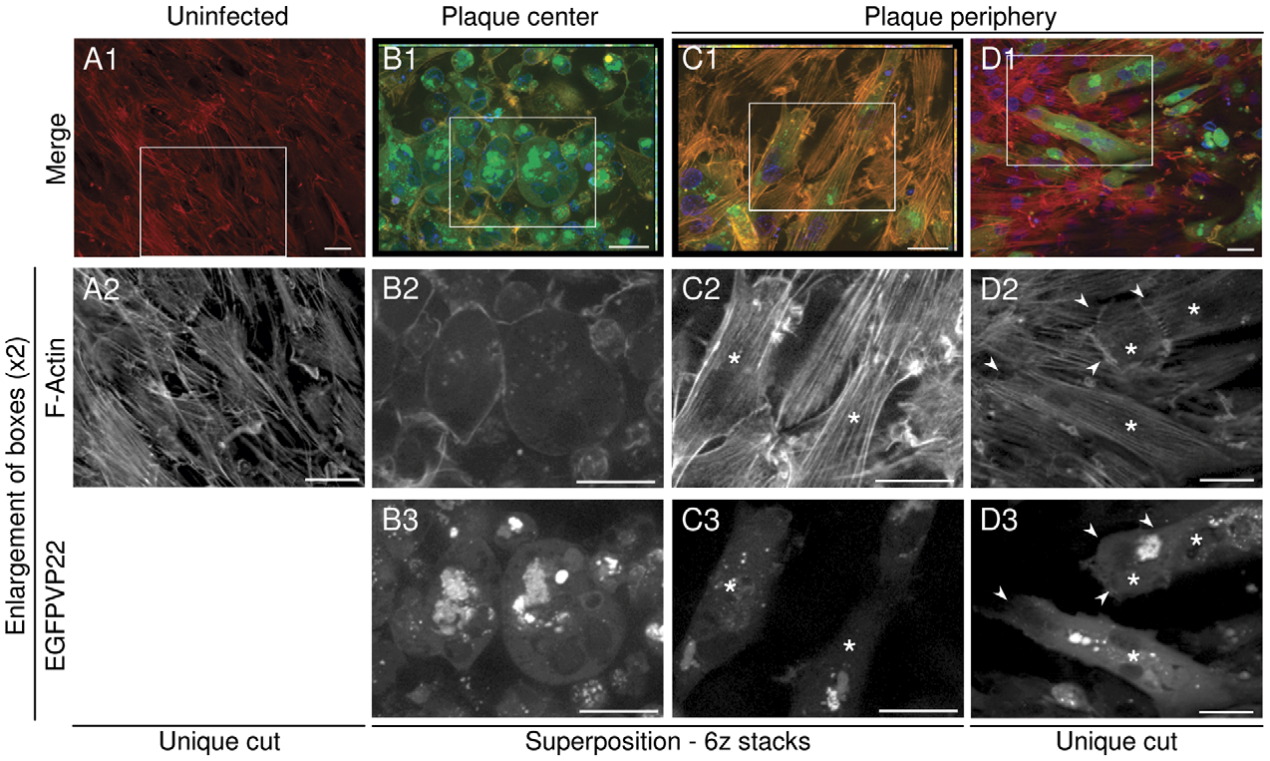
Actin cytoskeleton is rearranged in the center of MDV infection plaques but not in periphery. Immunofluorescence microscopy of non infected cells (column A) and BAC20-EGFP infected cells, after 4 days of growth on the CESCs monolayer are shown, in the center of a representative infection plaque (column B), or in the periphery (columns C–D). F-actin was stained with AlexaFluor594-Phalloidin (red) (line 2) and DNA with Hoechst 33342 (blue) (B1–D1). Infected cells express the EGFPVP22 protein (green) (line 3). Asterisks show infected cells still possessing stress fibers. Arrowheads show infected cells which present connections between their stress fibers and those of neighboring infected or uninfected cells. Note that pictures in columns B and C are a superposition of 6 z stacks of 0.250 µm each, whereas pictures A and D correspond to a unique cut. All scale bars, 20 µm.

MDV infection with the BAC20 strain in CEFs was described to destroy stress fibers in 70% of infected cells [33], when infected cells were reseeded sparsely. It appeared therefore important to us to analyze the actin cytoskeleton rearrangement directly at the level of an infection plaque, especially at its periphery where MDV cell-to-cell spread occurs. At high density, non-infected cells are well spread with a fusiform morphology, pronounced stress fibers mostly oriented in the long cell axis and cell-cell contacts, as expected for a cell preparation enriched in primary chicken skin fibroblasts (Fig. 2, A1–A2). At high density, cells often partially overlay especially at their extremities. Observation of BAC20-EGFP-infected cells localized in the center of a 4 day-old infection plaque showed that they are all devoid of stress fibers with reduced cell-cell contacts. Their morphology was also changed as they became round (Fig. 2, B1–B3). In contrast, infected cells localized at the peripheral rim of the viral plaque still conserved a substantial amount of stress fibers (Fig. 2, C1–D3) and cell-cell contacts. The morphology of these cells did not show pronounced changes compared to uninfected cells and we did not frequently observe the induction of long cell extensions from infected cells (not shown). Careful observations of peripheral infected cells also showed that their stress fibers are aligned with those of uninfected cells (Fig. 2, C1–D3). At the contact between two cells having their stress fibers in the same axis, the phalloidin staining was more pronounced with a zipper aspect shape, reminiscent of AJs, the only physiological cell-cell contact known in fibroblasts. Indeed, in such cell type, stress fibers arrive perpendicular to the cell-cell contacts where they anchor to AJs [24], [39], [40]. This feature could also be observed between two infected cells expressing the EGFPVP22 (Fig. 2, D1–D3). These results showed that stress fibers, the elongated shape of the cells and cell-cell contacts are maintained in infected cells at the periphery of viral plaques but not in their center. This also showed that stress fibers and cell-cell contacts are present until a late stage of infection (i.e. cells expressing EGFPVP22 tegument protein). This suggested that stress fibers and cell-cell junctions are destabilized only when cells are infected for a prolonged period of time and that spread could probably precede these profound cell changes.

### The inhibition of Rho and ROCK signaling decreases MDV spread

**Figure 3:**
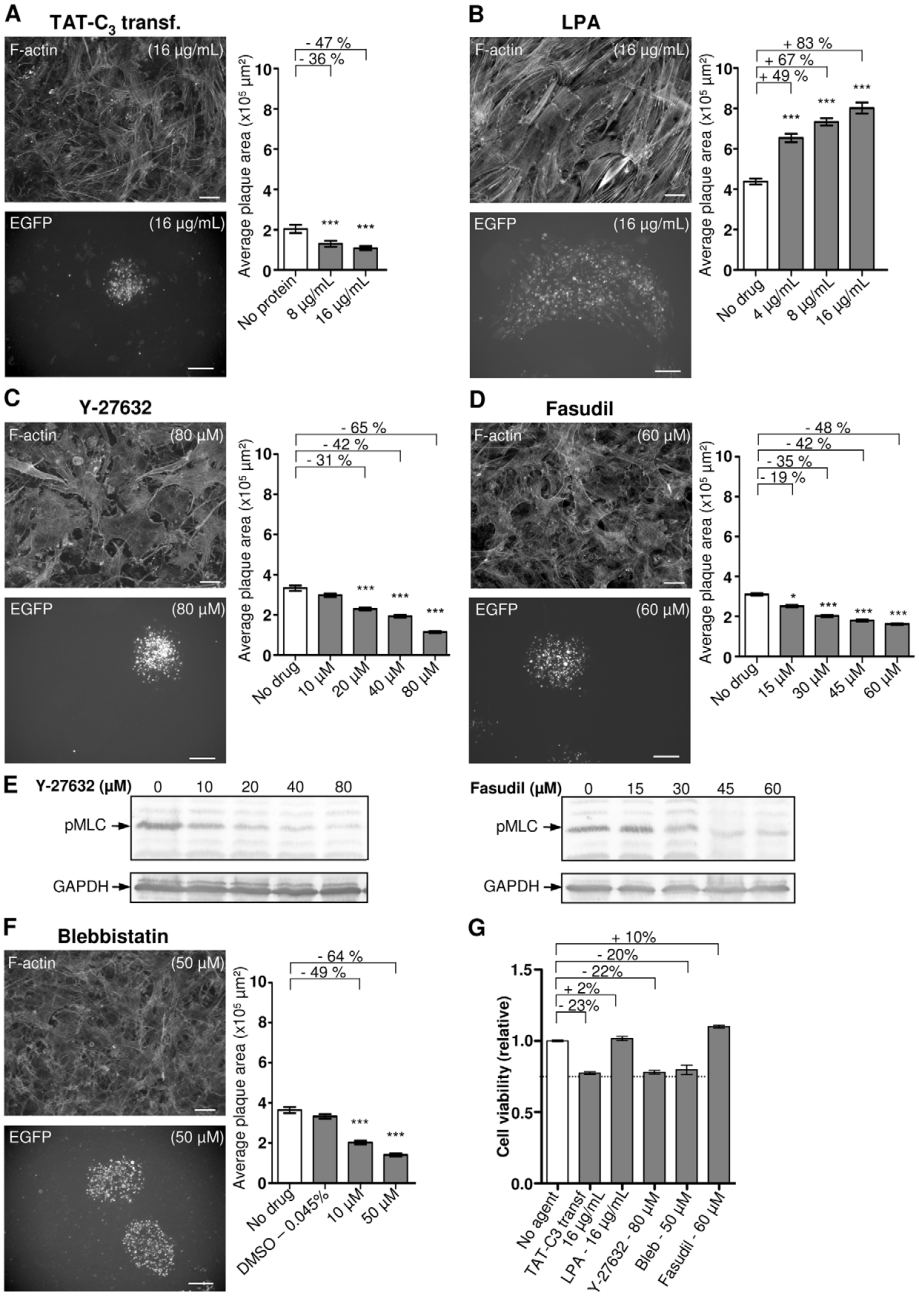
Inhibition of the Rho-ROCK signaling pathway decreases MDV cell-to-cell spread. (A) Effect of TAT-C3 transferase, an inhibitor of Rho proteins. Upper panel, F-actin staining of non-infected CESCs treated with 16 µg/mL of TAT-C3 transferase (bar, 20 µm). Lower panel, a picture of a representative BAC20-EGFP infection plaque obtained with 16 µg/mL of TAT-C3 transferase (bar, 200 µm). Right panel, the graph shows a quantitative analysis of plaques size in one of two to three representative experiments (***, p<0.001). The error bars represent the SEM of 50 plaques size. (B) Effect of LPA, an activator of Rho proteins, on F-actin (upper panel), plaques shape (lower panel) and plaques size (right panel), as in A (***, p<0.001). (C) Effect of Y-27632, an inhibitor of ROCKs, on F-actin (upper panel), plaques shape (lower panel) and plaques sizes (right panel), as in A (***, p<0.001). (D) Effect of Fasudil, a second inhibitor of ROCKs, on F-actin (upper panel), plaques shape (lower panel) and plaques size (right panel), as in A (*, 0.1<p<0.5; ***, p<0.001). (E) After a 4-day treatment with the indicated concentrations of Y-27632 (left) or Fasudil (right), MLC phosphorylation was monitored by Western blotting with an anti-phospho-MLC (Thr18/Ser19) antibody followed by an alkaline phosphatase-labeled secondary antibody. An anti-GAPDH antibody was used to control the amount of proteins in each lysate. (F) Effect of blebbistatin, an inhibitor of NMIIA ATPase activity, on F-actin (upper panel), plaques shape (lower panel) and plaques size (right panel), as in A (***, p<0.001). (E) One of two representative experiments showing the relative cell viability after a 4-day treatment with the highest concentration of TAT-C3 transferase, LPA, Y-27632, Fasudil and blebbistatin are presented. Values were measured and normalized as above. Error bars represent the SEM.

As Rho signaling pathway is well known to regulate stress fibers formation and maintenance, we next studied the impact of this pathway on MDV spread, by blocking or activating Rho protein activities. The transfection rate of primary CESCs barely exceeding 20–25%, we could not undertake approaches for which the success depends on high transfection rates of the cell layer, like RNAi or the overexpression of a dominant active or negative protein through an expression vector. We therefore use either drugs, receptor agonists or a TAT-purified protein. The TAT-C3 transferase is an ADP-ribosylating protein of *Clostridium botulinum* that inactivates the three different Rho proteins (A, B, and C) through direct covalent binding of an ADP-ribose to the asparagine 41 site [41], [42]. This purified protein system presents the advantage to target all cells [43] and to allow a control of the inhibitor quantity used, in contrast to the over-expression of a dominant negative mutant. The addition of 16 µg/mL of purified TAT-C3 transferase to the culture media of CESCs induced an incomplete stress fiber breakdown with cells remaining well spread (Fig. 3A). This phenotype, although moderate, indicated an effect on Rho, and showed indirectly that TAT-C3 transferase is able to penetrate through cell membrane to exert its biological activities in CESCs (Fig. 3A) as already described [43]. A more robust phenotype (like after microinjection) with a complete loss of stress fibers, a collapse of cell body and a decrease of cell spread would not have been compatible with our virus assay [44], [45]. In our conditions, MDV plaque sizes were decreased by 47% (Fig. 3A) and the cell viability was decreased by 23% (Fig. 3G), albeit the cell confluency was not altered. It should be noticed that TAT-C3 experiments were performed on 24-well plates whereas other experiments were done on 6-well plates and that for unknown reason the BAC20-EGFP plaque size was reproducibly approximately two times smaller in the former. Treatment of cells with 16 µg/mL of lysophosphatidic acid (LPA), a phospholipid known to activate Rho proteins activity through the LPA receptors, increased the size of plaques, up to 83% (Fig. 3B) without altering the cell viability (Fig. 3G).

The two major effectors of Rho proteins are ROCKs and Dia involved in actomyosin contraction and actin filament polymerization, respectively [27]. Due to the abundance of stress fibers in CESCs cell monolayers under normal conditions, and the lack of tools for studying chicken Dia, we examined solely the involvement of ROCK proteins. To this end, we used the Y-27632, reported to specifically inhibit the kinase activity of ROCK 1 and 2, without inhibiting those of PAKs or MLCK [46]. As expected, CESCs treatment with 80 µM of Y-27632 for 4 days led to a loss of stress fibers (Fig. 3C). This treatment also led to cell extensions induction and gaps between cells suggesting that cell-cell junctions are disrupted as previously reported [24]. Y-27632 treatment also resulted in decreased MDV plaque sizes in a dose-dependent manner with a 65% reduction at the highest concentration used (Fig. 3C). In presence of 80 µM of Y-27632, a reduction of 22% in CESCs viability was measured (Fig. 3G). To confirm ROCK contribution, we used a second ROCK inhibitor, the Fasudil (also named HA 1077). This compound was reported to inhibit the kinase activity of ROCKs as well as other kinases in particular at high concentrations [47], [48]. This molecule, like Y-27632, induced breakdown of stress fibers and of cell-cell junctions as well as cell shape changes (Fig. 3D). The Fasudil also reduced MDV plaques size in a dose-dependent manner with a 48% reduction at the highest concentration used of 60 µM. At this dose, no reduction in CESCs viability was recorded. The viability was even increased compared to untreated conditions suggesting a stimulation of the cell division (Fig. 3G). Therefore both ROCK inhibitors decreased MDV in a dose dependant manner. In order to evaluate the possible off-target contribution of ROCK inhibitors in the MDV spread especially at high doses, we next analyze the phosphorylation of a major ROCK substrate, the Myosin Light Chain (MLC) in presence of Y-27632 and Fasudil in CESCs. Monolayers of non-infected CESCs treated with different doses of Y-27632 or Fasudil for 4 days were lyzed and the lysates analyzed by Western blotting using an antibody which recognize phosphorylated MLC. Despite high background with the anti-phospho-MLC on chicken cell lysates, a quasi total inhibition of MLC phosphorylation was observed with Y-27632 between 20 and 40 µM and with Fasudil between 30 and 45 µM (Fig. 3E). These concentrations are in accordance with the ones previously used in rat and chicken embryonic fibroblasts [46], [49]. This result indicates that the supplemental decrease of MDV spread observed at upper dose of Y-27632 and Fasudil are probably due to off-targets effects. All together, these results show that MDV spread is dependent on ROCKs activity.

The Rho-ROCK pathway promotes actomyosin II contraction via the direct phosphorylation of MLC phosphatase and MLC via ROCKs [31]. We therefore investigated the role of actomyosin II contraction on MDV cell-to-cell spread. For that, we used blebbistatin (Bleb), a specific inhibitor of the non muscular myosin II (NMII) ATPase activity [50]. Chicken cells treated with 50 µM of Bleb showed a partial disorganization of stress fibers (Fig. 3F) as well as small gaps between cells suggesting a loosening of cell-cell junctions. This cell phenotype indicated an action on avian cells similar to the one described in mammalian cells [24], [51]. Treatment of the CESCs monolayer with 50 µM of Bleb yielded a 64% reduction of MDV spread (Fig. 3F) with a cell viability reduction of 20% (Fig. 3G). Thus, Rho-ROCK activity and actomyosin II contraction, a final effector of this signaling pathway appears to be crucial for MDV cell-to-cell spread on primary CESCs.

### Rac-PAK signaling have an inhibitory effect on MDV cell-to-cell spread in CESCs

**Figure 4:**
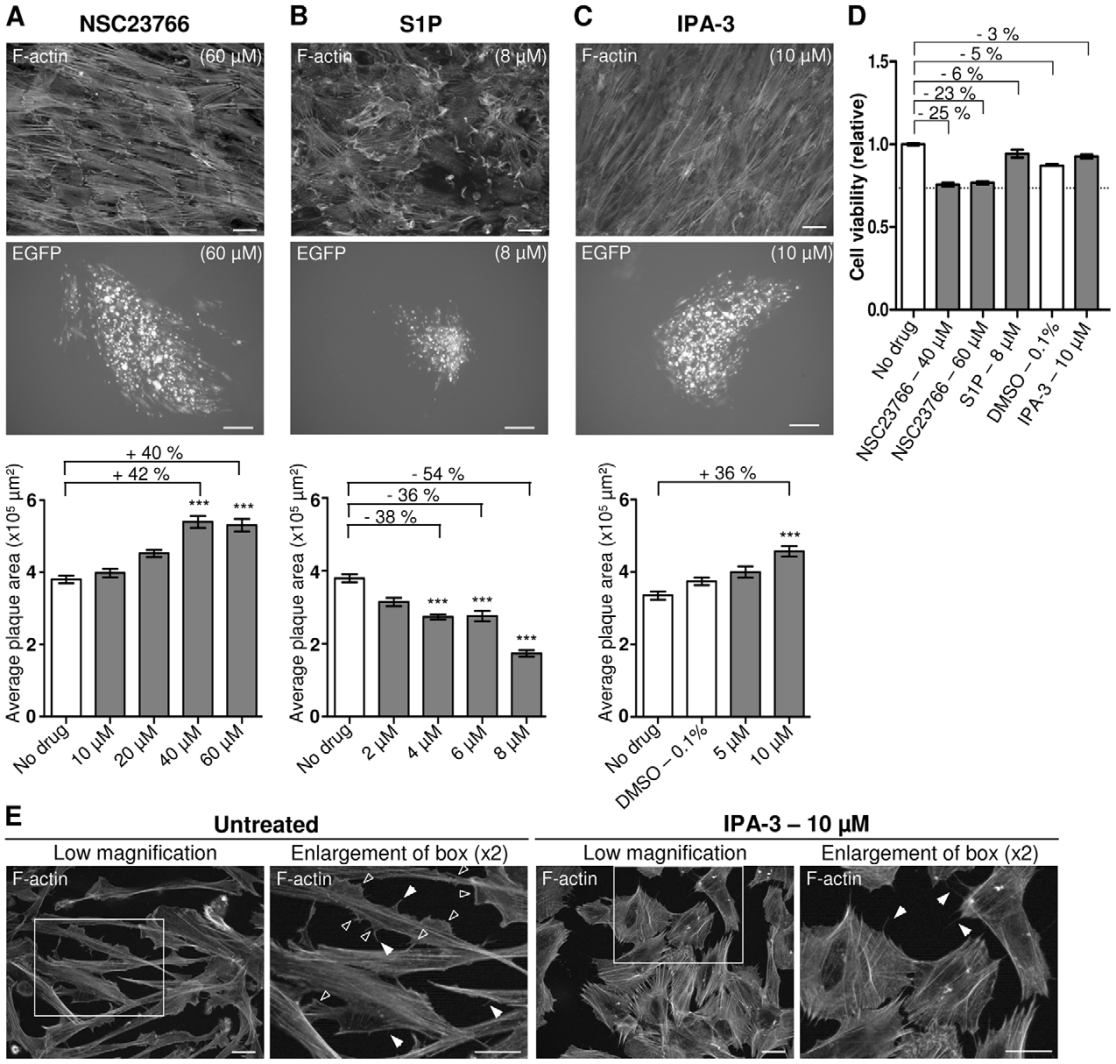
MDV cell-to-cell spread is enhanced upon inhibition of the Rac signaling pathway. Monolayers of CESCs were infected or not with the BAC20-EGFP for 6 hrs, and then treated for 4 days with the indicated concentrations of inhibitors or activator. (A) The effect of a Rac inhibitor, NSC23766, on MDV cell-to-cell spread. Upper panel, F-actin staining of non-infected CESCs treated with 60 µM of NSC23766 (bar, 20 µm). Middle panel, a representative BAC20-EGFP infection plaque obtained following treatment with 60 µM of NSC23766 (bar, 200 µm). Lower panel, the graph shows a quantitative analysis of plaques size in one of three representative experiments. Percentages on graphs indicate the increase (+) in plaques size compared to the untreated cells (***, p<0.001). The error bars represent the SEM of 50 plaques size. (B) The effect of S1P, a Rac1 activator, on F-actin (upper panel), on plaques shape (middle panel) and plaques size (lower panel), as in A (***, p<0.001). (C) Effect of IPA-3, an inhibitor of group I-PAKs, on F-actin (upper panel), plaques shape (middle panel) and plaques size (lower panel), as in A (***, p<0.001). (D) One of three representative experiments showing the relative cell viability after a 4-day treatment with the highest concentration of NSC23766, S1P and IPA-3 are presented. Values were measured and normalized as presented above. Error bars represent the SEM. (E) Control of IPA-3 on CESCs. F-actin was stained on sparsely seeded CESCs, untreated (left), or treated for 2 hrs with 10 µM of IPA-3 (right). For each condition, a two fold magnification of the box is shown next to the lower magnification. Solid arrowheads point to filopodia while open arrowheads point to lamellipodia. All bars, 20 µm.

Given the apparently importance of Rho-ROCK signaling in MDV cell-to-cell spread, we next wanted to confirm these results by analyzing the effect of Rac-PAK signaling. Indeed, Rac is known to counteract stress fibers formation through its inhibitory action on MLCK via PAKs in many systems, including primary chicken embryo fibroblasts [25], [52], [53]. Rac1 involvement was examined using two agents: the NSC23766, a molecule which was described to specifically inhibit Rac1 by preventing the binding between Rac1 and its activator proteins, the guanosine nucleotide exchange factors [54] and sphingosine-1-phosphate (S1P), a lipid that activates Rac1 through S1P-receptor signaling [55]. A treatment of CESCs with 60 µM of NSC23766 during the MDV spread assay resulted in a 40% increase of MDV plaque sizes (Fig. 4A). Conversely, MDV spread was decreased by 54% when the cell monolayer was treated with 8 µM of S1P (Fig. 4B). S1P treatment reduced cell viability by 6% while treatment with NSC23766 reduced it by up to 25%, at the highest concentrations (Fig. 4D). Despite a reduction in cell viability, the cell monolayer remained confluent after NSC23766 treatment and MDV spread was increased.

Having shown a negative effect of Rac signaling on MDV dissemination, we then tested the effect of PAKs, downstream effectors of Rac1. To this end we used IPA-3, a specific inhibitor of group I-PAKs, which does not affect ROCKs or MAP kinase proteins [56]. IPA-3 activity was controlled on the basis of lamellipodia disappearance in CESCs plated at low density (Fig. 4E). An IPA-3 concentration of 10 µM specifically increased the MDV plaque size by 36% (Fig. 4C) while cell viability remained virtually unaffected (Fig. 4D).

All together, these results show that MDV dissemination is negatively regulated by Rac1 stimulation involving group-I PAKs effectors signaling and is promoted by Rac1 inhibition.

### LPA and S1P effects on MDV spread are totally or partially mediated by Rho and Rac signaling

**Figure 5:**
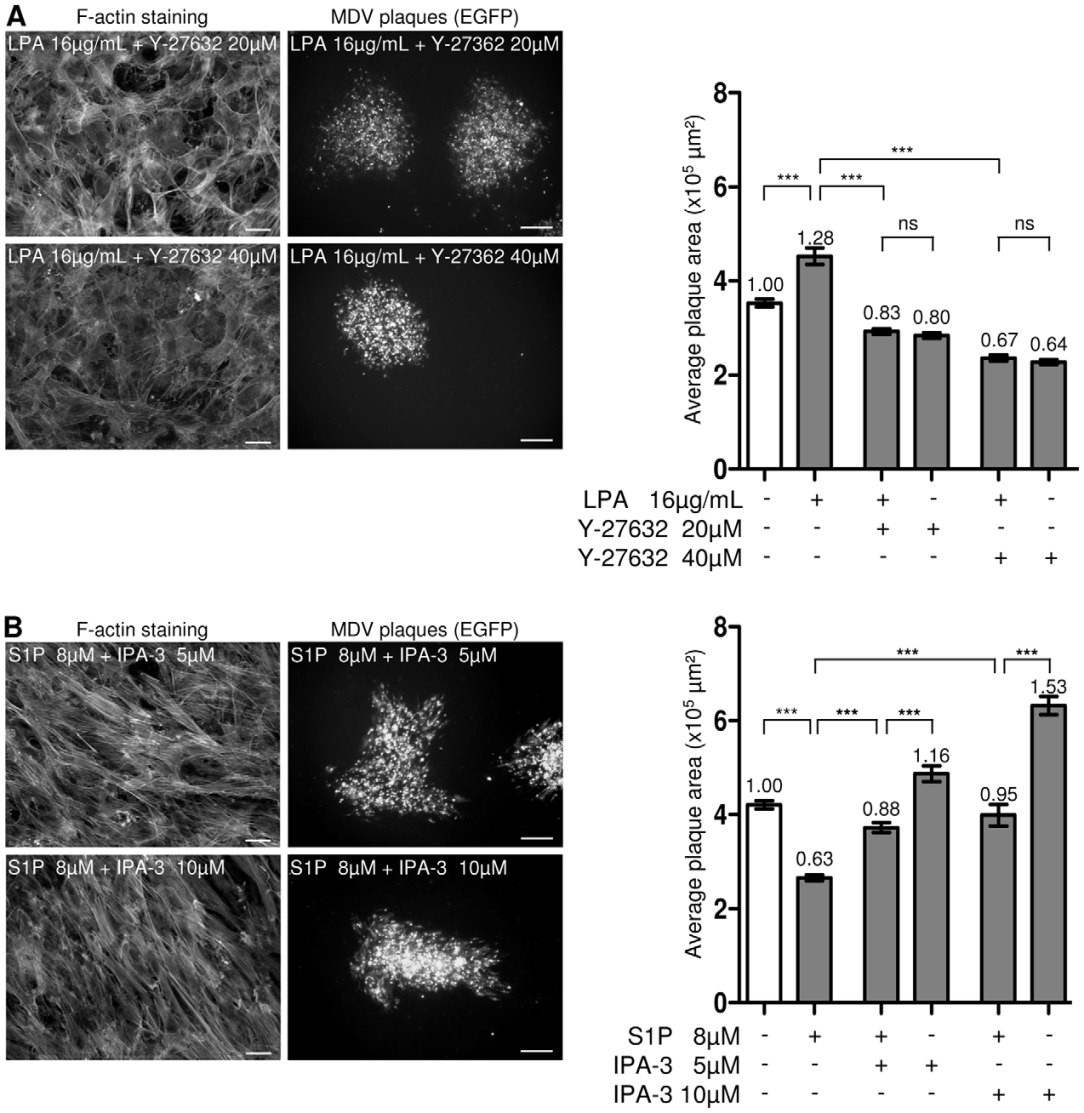
LPA and S1P effects on MDV cell-to-cell spread are mediated through Rho-ROCK and Rac-PAK respectively. Monolayers of CESCs were infected or not with the BAC20-EGFP for 6 hrs, and then treated for 4 days at the indicated molecule concentrations. (A) Specificity of the LPA effect on MDV spread through the Rho-ROCK pathway. In the left panel, a representative picture of both F-actin staining of non-infected CESCs (bar, 20 µm) and BAC20-EGFP infection plaque (bar, 200 µm) obtained after each double treatment. In the right panel, the graph shows a quantitative analysis of plaques size in one representative experiment over two (ns, non significant; ***, p<0.001). The number at the top of each histogram indicates the ratio between plaques size after treatment compared to the untreated condition. The error bars represent the SEM of 50 plaques size. (B) Specificity of the S1P effect on MDV spread through the Rac-PAK pathway. The effect of the indicated double treatments on F-actin and on the plaques size are presented like in A.

In order to investigate if LPA and S1P may influence MDV spread through other signaling pathways than Rho-ROCK and Rac-PAK respectively, we examined MDV spread in presence of these activators in combination with a selective inhibitor acting downstream of each signaling pathway. We first measured MDV plaques size in presence of 16 µg/mL of LPA in combination with two concentrations of Y-27632 (20, 40 µM), inhibiting partially or totally MLC phosphorylation and not modifying cell viability. In these conditions, MDV spread was not significantly different from the one measured with Y-27632 alone at the same concentration (Fig. 5A). This suggests that LPA effect on MDV spread is totally counteracted by Y-27632 and is preferentially mediated by ROCKs. We next examined MDV plaque size, after simultaneous treatment with S1P at 8 µM and IPA-3 inhibitor (5 or 10 µM). The use of 10 µM of IPA-3 in presence of 8 µM of S1P restored MDV plaque size similar to untreated condition (Fig. 5B). Nevertheless, MDV spread did not reach the level obtained in presence of IPA-3 alone (Fig. 5B). This result indicates that S1P effect is partially counteracted with type I-PAKs inhibition and can be interpreted in three manners: (i) either 8 µM S1P activates more PAKs molecules via Rac than IPA-3 at 10 µM is able to inhibit (ii) S1P may activate PAKs isoforms not inhibited by IPA-3, (iii) or S1P activates another pathway than Rac-PAK, having a negative effect on MDV spread. All three hypotheses are plausible as (i) for a fixed concentration of S1P, the plaque size responds to IPA-3 in a dose-dependent manner, (ii) IPA-3 only inhibits type I-PAKs and we cannot exclude a role for other PAKs isoforms activated by S1P [56] and (iii) S1P can also signal through other pathways, like PI3K/akt and Ras/Erk, which may affect MDV spread independently of PAKs [57].

### MDV plaques are shaped in relation with the cell shape and the semi-ordered cell arrangement in its micro-environment

**Figure 6:**
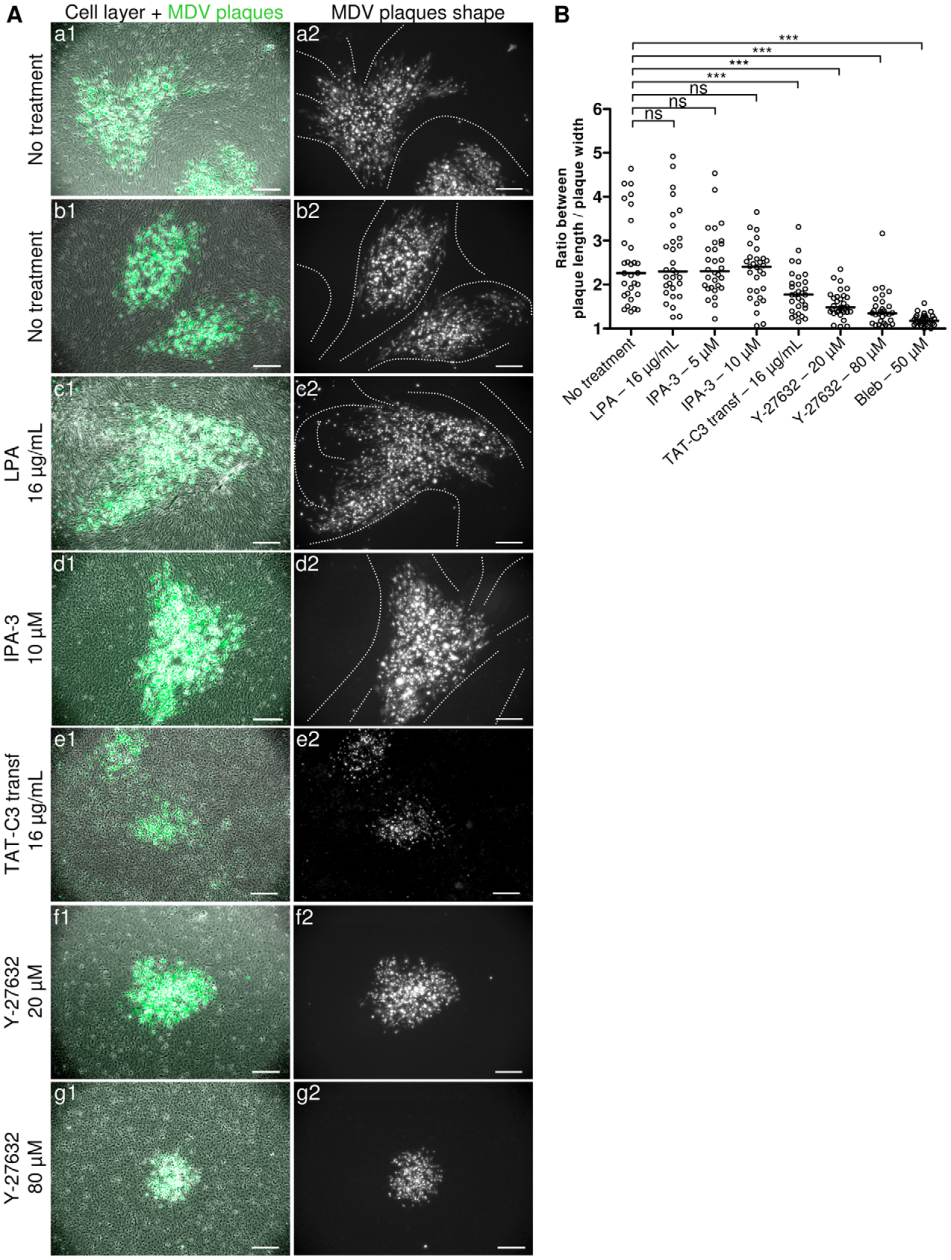
Inhibition of Rho-ROCK signaling results also in a rounding of MDV plaques. (A) Typical images of BAC20-EGFP infection plaques 4 days post-infection without treatment (lines a and b), or in the presence of 16 µg/mL of LPA (line c), 10 µM of IPA-3 (line d), 16 µg/mL of TAT-C3 transferase (line e), 20 µM of Y-27632 (line f) or 80 µM of Y-27632 (line g) are shown. For each condition, the cell monolayer was photographed in white light with phase contrast and infected cells in fluorescence with EGFPVP22. Cell monolayer (greyscale) and infection plaques (green) are shown merged (column 1). In some conditions, a semi-ordered cell arrangement is visible in uninfected region of the cell monolayer with phase contrast. This cell arrangement is indicated with white dashed lines on the pictures (column 2). Bars, 200 µm. (B) The graph shows a quantitative analysis of the ratio between plaque length and plaque width under the indicated growth conditions. A ratio of 1 corresponds to a perfect circle. The ratio will be increased by the degree to which the plaque is elongated. The median value for each experimental group is indicated by a horizontal bar. (***, p<0.001).

In order to find to which cell structure linked to stress fibers MDV cell-to-cell spread could be related, we carefully inspected the BAC20-EGFP-infected cell monolayers by phase contrast microscopy (Fig. 6A) and by fluorescence microscopy after phalloidin-labelling of F-actin (not shown). Mock-infected cells and cells outside of viral plaques are tightly packed, joined, spindle-shaped with their elongated body generally in line with the one of neighbouring cells, in a semi-ordered manner highlighted with white dashed lines in Fig. 6A. Moreover in these cells, the central stress fibers are generally oriented with the long axis of the elongated body and therefore in relation with the cell shape (Fig. 2A). In normal growth conditions, the MDV plaques were pleiomorphic, irregularly shaped, appearing to follow the pattern of surrounding semi-ordered cells (Fig. 6A, lines a and b). It is noticeable that when several plaques are next to each other, they may show a parallel orientation (Fig. 6A, line b). This observation shows that MDV plaques shape is related to the cell shape and arrangement of uninfected surrounding cells in its environment.

### Inhibition of Rho-ROCK signaling results in plaques morphology change in addition to plaques size reduction

We next explore if the plaque morphology was modified in presence of molecules modulating Rho/Rac pathways. In the presence of 16 µg/mL of LPA or of 10 µM of IPA-3 (Fig. 6A, lines c and d) as well as with NSC23766 (data not shown), the conditions in which stress fibers remained intact as well as cell shape and cell arrangement at the monolayer level, the overall plaques morphology was pleiomorphic, like in control condition. Interestingly, when actin-depolymerizing agents were used, including TAT-C3 transferase at 16 µg/mL (Fig. 6, line e), Y-27632 at 20 µM or 80 µM (Fig. 6, lines f and g) and Bleb at 50 µM (data not shown), stress fibers and pattern of semi-ordered cells disappeared. In these conditions, MDV plaques adopted a more rounded shape, in addition to displaying a reduced size. The morphological differences in plaques shape were supported by an analysis of the ratio between plaque length and plaque width. These ratios were closer to 1 with TAT-C3 transferase, Y-27632 and Bleb compared to untreated conditions, what was expected for more rounded plaques (Fig. 6B). In contrast, no difference in these ratios was observed with LPA at 16 µg/ml or IPA-3 at 5 or 10 µM (Fig. 6B), conditions in which the plaques shapes remained pleiomorphic with more elongated and variable forms.

In conclusion, these results indicate that the semi-ordered arrangement of the cells has a marked influence on the shape of MDV plaques. Moreover, when the semi-ordered arrangement of the cells was abrogated by stress fibers depolymerization, MDV plaques became more circular and isotropic. These observations suggest that the MDV infection may progress preferentially from one cell to another in plaques periphery, when their stress fibers are matched in a row.

### Rho/Rac signaling pathways involvement in MDV spread is independent of the viral strain used

**Figure 7:**
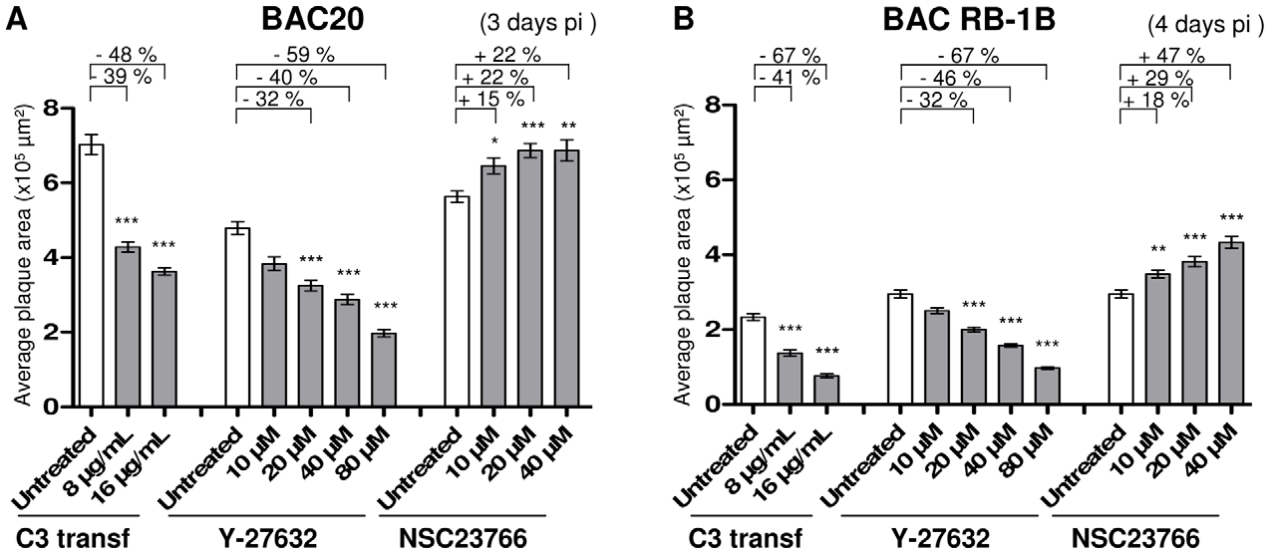
Opposing effects of Rho/Rac pathways in MDV cell-to-cell spread is conserved for two MDV strains. Monolayers of CESCs were infected or not with one of the two following viruses: (A) the attenuated parental strain BAC20 and (B) the virulent wild-type BAC RB-1B. Six hrs post-infection, one of the three agents, TAT-C3 transferase, Y-27632 or NSC23766, was added to the cells until the end of the spread assay. The spread assay lasted 4 days for BAC RB-1B and 3 days for BAC20, due to its fast growth. The graphs show a quantitative analysis of infection plaques size for each virus in one of two representative experiments (***, p<0.001; **, 0.001<p<0.01; *, 0.01<p<0.05).

So far we presented spread data obtained with a recombinant MDV reporter having the N-terminus of VP22 fused to EGFP that is partially impaired in its spread efficacy compared to the parental BAC20 virus. In order to verify that our previous observations were not specific of this mutant and to discard a potential association with VP22, we next assayed two parental recombinant viruses: the non-pathogenic BAC20 and the highly virulent recombinant BAC RB-1B strain [58]. It should be indicated here that the BAC RB-1B, which is highly virulent in chickens, produces smaller plaques than the non-pathogenic BAC20, adapted to cell culture. The plaques size obtained with the BAC RB-1B are in the same range than those obtained with the BAC20-EGFP on CESCs. Spread of both parental viruses was dose-dependently diminished when TAT-C3 transferase or Y-27632 was added to the CESCs culture media (Fig. 7). Moreover, the presence of NSC23766 led to an increased dissemination for these two viruses, as previously seen with the BAC20-EGFP. Lastly, the same influence of the semi-ordered cell arrangement on plaques shape was observed with the BAC RB-1B and BAC20 viruses (data not shown).

These data clearly show that the spread of the two parental viruses is affected in the same manner by Rho/Rac pathways manipulation than the BAC20-EGFP, independently of the strain origin and of the original spread efficiency. They also demonstrate that Rho/Rac signaling pathways regulates MDV independently of its tag fused to VP22.

### Rho-ROCK pathway inhibition and the concomitant stress fiber depolymerization are associated with the disappearance of adherens junctions in CESCs

We next examined the AJs presence and stability in CESCs after Rho/Rac modulations for several reasons: (i) the presence of cell-cell junctions reminiscent of AJs in our cell layer between infected and non-infected cells (Fig. 2), (ii) the anchorage of stress fibers ends to AJs in fibroblasts [24], [39], [40], (iii) the previous description of a role for AJs into HSV-1 cell-to-cell spread in polarized epithelial cells [10] and (iv) the important role of Rho signaling in the formation and/or maintenance of AJs in mammalian myoblasts or fibroblasts [24], [59], [60]. The cell junctions were re-examined at high magnification after phalloidin staining of monolayers of non-infected cells treated for 4 days with a panel of molecules affecting the Rho/Rac pathways. The zipper aspect shape at the cell junctions was visible under all conditions where MDV spread was not modified (untreated cells) or increased (LPA, NSC23766) (Fig. 2A, 3B and 4A). It is noteworthy that the cell junctions appeared more pronounced after treatment with NSC23766 or LPA, two conditions under which MDV spread was increased (Fig. 3B and 4A). In contrast, the zipper aspect shape at the cell contacts was partially or totally lost in conditions where MDV cell-to-cell spread was decreased, following treatment with TAT-C3 transferase, Y-27632, Fasudil or Bleb (Fig. 3A, 3C, 3D and 3F).

**Figure 8:**
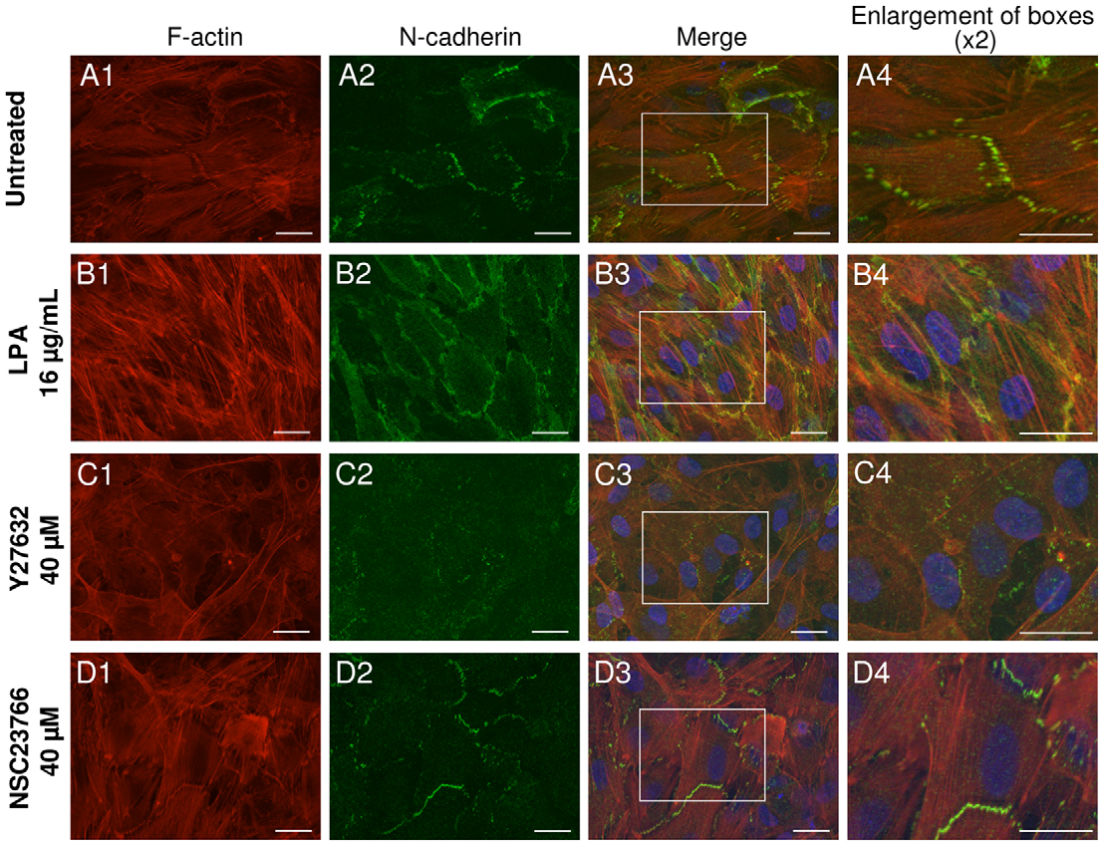
Rho/Rac pathways have opposing effects on the accumulation of N-cadherin at cell-cell contact zones. Typical images of adherens junctions observed by staining of N-cadherin are shown. Before staining, the CESCs monolayer was left untreated (line A), or treated for 4 days with 16 µg/mL of LPA (line B), 40 µM of Y-27632 (line C) or 40 µM of NSC23766 (line D). DNA was stained with Hoechst 33342 (blue), F-actin was stained with AlexaFluor594-Phalloidin (red) (column 1) and adherens junctions were stained with an anti-N-cadherin and an AlexaFluor488 secondary antibody (green) (column 2). Bars, 20 µm.

In order to better characterize cell junctions in CESCs, a confluent CESCs monolayer was stained for F-actin and for N-cadherin, a specific component of AJs in fibroblasts and myoblasts [59], [61], [62]. We observed that under normal conditions, N-cadherin was localized exclusively at cell contacts between two aligned cells as expected for an AJs marker in such cells (Fig. 8, A2). This staining appeared fragmented as dots between two cells and located preferentially when their stress fibers are in line with each other at both ends of these junctions (Fig. 8, A3–A4). We next studied the N-cadherin and F-actin stainings in cells treated with agents, which act on the Rho/Rac pathways and thereby modulate MDV spread. With 16 µg/mL of LPA, the N-cadherin staining was more evenly distributed at the cell periphery and less discontinuously (Fig. 8, B2–B4). In addition, the stress fibers appeared thicker and less parallel than under the control conditions (Fig. 8, A1 and B1). In the presence of 40 µM of Y-27632, the N-cadherin staining almost disappeared as well as the central stress fibers (Fig. 8, C1–C4). Finally with 40 µM of NSC23766, the N-cadherin preferentially localized when their stress fibers are in line with each other at both ends of these junctions, like under control conditions (Fig. 8, D1–D4). The major difference is that the N-cadherin staining at the cells junctions looked continuous and not fragmented. Medium doses of Y-27632 and NSC23766 were used in this experiment as they give good ratio of MDV spread on cell viability. Taken together, these results show that Rho/Rac pathways modify the N-cadherin presence at the cell-cell junction of CESCs in an opposite manner.

## Discussion

The ability of MDV to spread from cell-to-cell is essential for MDV multiplication in cell culture systems and in its chicken host. The objective of this work was to examine this process in relationship with actin filaments and RhoGTPases that regulate their organization and dynamic as well as actin-associated structures.

Initially we verified in our cell culture system, enriched in embryonic skin fibroblasts, the importance of F-actin and actin dynamic for MDV spread. CytD which prevents actin polymerization led to a drastic reduction of 69% in MDV spread as reported previously [63]. Jaspl, considered as an actin-stabilizing toxin *in vivo* and *in vitro* also induced a decrease in MDV spread but less marked (31%). A reduction in cell-to-cell spread was also observed with Jaspl for PRV in sparsely plated swine testis cells [8]. Both drugs were difficult to handle, as CytD also completely inhibits cell adhesion at higher concentration and Jaspl prevents actin depolymerization but also induces actin polymerization [36]. We were indeed able to detect actin aggregates as previously described for mammalian cells [37]. With respect to Jaspl, the reduction of MDV spread can be explained in at least three ways. Firstly, that normal F-actin dynamics contribute to MDV spread. Secondly, it is conceivable that the observed defect in MDV spread is associated to the “indesirable” actin polymerizing activity of Jaspl. Indeed, it can be reasoned that abnormal actin-containing structures may trap cytoplasmic particles and subsequently reduce the efficiency of virus egress and spread, a hypothesis hardly verifiable for MDV. Lastly, Jaspl could also stabilize cortical actin and makes this barrier more difficult to cross by the virus at the entry or the exit step.

We showed a role of Rho/Rac signaling pathways on MDV cell-to-cell spread by using a panel of chemical inhibitors and purified biological receptor agonists or toxin. Such molecules could have off-target effects which may also possibly modulate MDV cell-to-cell spread. Our results obtained with double treatments (S1P plus IPA-3) indicate that other pathways may indeed contribute to the MDV spread modulation, but confirmed that S1P effects on MDV spread is partially mediated through type I-PAK. Such off-target effects were not detected with LPA on the Rho-ROCK pathway in our assay. Other results also indicates that off-targets may not be responsible alone for the MDV spread modulation observed with the different pharmacological molecules: (i) two ROCK inhibitors produced a comparable MDV spread reduction, (ii) activators and inhibitors of a cellular target (either Rho or Rac) gave opposite effects despite acting with different mechanisms, (iii) all inhibitors targeting proteins involved at different levels in the Rho pathway led to a similar decrease in MDV spread, whereas the opposite effects were obtained with Rac inhibitors. Therefore, although off-targets effects could contribute to some of the MDV spread modulations observed, especially at the highest doses (eg. for ROCKs inhibitors), all our results support a role of Rho/Rac in MDV spread.

Rho/Rac signaling pathways were described to be associated to the cell-to-cell spread of herpesviruses, like the MHV-68, PRV and HSV-1. For these viruses, the reported mechanisms were in relation to the development of cell extensions containing actin filaments at late stages of infection. For the MHV-68, fibroblastic NIH-3T3 cells developed membrane fronds with multibranched cell extensions 16 hrs pi. This membrane remodeling required the gp48/ORF58 that promotes intercellular spread and is highly dependent on RhoA but only moderately dependent on Rac1 [9]. For PRV, infected ST or RK13 cells plated at low density developed long cellular projections containing actin and microtubules, 4 to 6 hrs pi, and fluorescent capsids can move inside toward their tips [8]. These cell projections were associated with US3 protein expression [8] and to be PAK1 dependent, with US3 protein directly phosphorylating group I PAKs [64]. Our data argue against a model in which MDV does also induce and use such cellular extensions as a major route for its spread in CESCs. Firstly, we did not observe an induction of extensive cell projections by fluorescence microscopy either on cell-sorted infected CESCs plated at low density [3] or in the periphery of plaques under normal conditions herein. Secondly, no overexpression of MDV viral protein was described to induce the formation of membrane cell projections including US3 protein in primary chicken embryo fibroblasts [63]. The fact that in our study the inhibition of PAK activity did not reduce MDV cell-to-cell spread but increased it, also argues against a role of group I PAK-mediated formation of cellular extensions in this process.

Cell-to-cell spread efficiency is the result of the virus cell-to-cell spread itself and of the virus cycle from the entry to the assembly of infectious virions, and probably not as a simple sum of virus release and entry [5]. Since several years, growing evidences show the role of actin and various RhoGTPases during early and/or late steps of herpesviruses cycle as thoroughly reviewed [65], [66], [67], [68], [69]. Therefore for MDV, as cell-to-cell spread cannot be separated from replication, entry and egress need also to be taken into account in the interpretation of the cell-to-cell spread results. We are now discussing if one of these steps could explain the favorable effect of Rho-ROCK pathway and the negative one of Rac-PAK that we observed in MDV spread. With respect to entry, for other herpesviruses the involvement of RhoGTPases was always evaluated a short time post-infection with cell-free virions at moderate moi, experimental conditions which are significantly different from ours (infection at low moi with cell-associated virus, long duration of infection with multiple virus cycles, on a dense cell layer). Among theses studies, a few depicted a positive effect of RhoA or ROCKs on the early stages of infection [70], [71], [72], [73]. For HSV-1, the entry in primary human corneal fibroblasts and nectin-1-CHO cells was described to involve RhoA activation and to be mediated by a phagocytosis-like uptake RhoA-dependent [70]. This process requires, before RhoA activation, the formation of membrane ruffles which depends on Rac1 and PAK1 [70], [74], a pathway that is not favorable for MDV cell-to-cell spread. This led us to conclude that a phagocytosis-like uptake in CESCs cannot explain by itself the effect of Rho/Rac signaling on MDV spread. Another example is EHV-1 [71], which enters via an endocytic mechanism or by direct fusion at the cell surface according to the cells, like HSV-1. In both situations, EHV-1 requires ROCK activity for a productive EHV-1 infection through a non-elucidated mechanism, as demonstrated in presence of Y-27632 or after overexpression of Gem, a negative ROCK1 regulator. A similar mechanism is therefore possible for MDV. The last example concerns KSHV, which was found to activate RhoA early after infection in human fibroblast cells, with RhoA having a role in the nuclear delivery of viral DNA [72]. Here the role of RhoA was associated to Dia2, another of its downstream effector [73] and the MTs stabilization, a mechanism which cannot be excluded at that time for MDV. The role of RhoGTPases at late stages of infection was poorly studied compared to entry. Here again, we should consider a positive role of Rho-ROCK and actomyosin contraction during these steps, either promoting viral particles intracellular trafficking or exocytosis of infectious virions. About transport, it is unlikely that the stress fibers could be used as tracks in the cytoplasm for capsids movement, as such mechanism was never reported to our knowledge for long distance movement of viruses. About exocytosis, it is worthwhile to mention in this context that NMII was reported as being involved in HSV-1 secretion [75]. However, this conclusion is based on the interpretation that BDM is a NMII inhibitor, a property that has been disputed in the literature [76], [77]. Nevertheless, we cannot totally exclude an NMII contribution to MDV release as NMII was reported to be involved in constitutive exocytosis and in Ca^2+^-trigged release of secretion granules [78]. This hypothesis cannot be tested because MDV extracellular virions are not detectable in cell culture. Therefore at this stage, a promoting role of Rho-ROCK during entry and/or of NMII activity during the late stages, although not testable, are possible mechanistic hypotheses.

In this study, we demonstrate an opposing influence of Rho/Rac pathways on MDV cell-to-cell spread with Rho-ROCK signaling positively regulating it. Whether MDV infection directly activates Rho and/or suppresses Rac signaling or whether the MDV has just benefited from the physiological Rho/Rac balance already present in non-infected cells for its replication and spread has not been assessed. Indeed two limitations combine to address this question: (i) only a fraction of the cells are infected and from various time and (ii) only a small fraction of the total Rho pool is activated at the same time in a cell (5%) [79]. Therefore, answering to this question will be very difficult for MDV in absence of cell-free inoculum to truly synchronized infection and performed it at moderate moi at a define post-infection time point. The first hypothesis, that MDV infection modifies Rho/Rac signaling at one step of its virus cycle, is likely as many herpesviruses do so [65], [69], even if the absence of pronounced cell morphology changes and actin cytoskeleton reorganization in infection plaque periphery, assumed to contain the more recently infected cells, does not suggest so. Nevertheless, these changes could be to short in time to be noticeable without synchronization of infection. The second hypothesis, that MDV just exploits the physiological Rho-ROCK activity for its replication and spread must be considered and cannot be ruled out. Indeed, our non-infected cell cultures exhibit a typical Rho phenotype with large number of stress fibers and focal adhesions plaques (not shown).

Here we collected data supporting a model in which stress fiber maintenance associated to cell shape and cell-cell junction persistence between infected and non-infected cells is critical for MDV cell-to-cell spread. Firstly, we observed that stress fibers are still present in infected cells at the periphery of the viral plaque. *Prima facie*, the persistence of stress fibers in infected cells was surprising as the virus encodes a US3 protein described to breakdown stress fibers. However, this feature was observed only in less than 70% of infected cells [63], an observation that is in good agreement with our own data. Moreover, with a *US3* deleted MDV, stress fibers breakdown was still observed in 50% of the infected cells [63]. Taken together these observations suggest that the stress fibers disappearance in MDV infected cells is only partially due to US3 protein and is possibly more a reflection of the cytopathogenic effect. Secondly, we have shown that under conditions favoring stress fiber preservation and elongated shape, the plaque morphology was linked to the semi-ordered arrangement of the cell in the plaque microenvironment. In contrast, when stress fibers formation was inhibited and elongated shape was lost, the plaque morphology was altered towards a more circular and smaller appearance. This suggests that stress fibers possibly in association with cell shape could influence the direction of viral spread and contribute to its efficiency. In addition, a possible role of the Golgi may be evocated. Indeed, when cells are cultivated on substrate with extracellular matrix micropatterns, intracellular trafficking out of the Golgi is oriented toward cell adhesive regions [80]. As adhesive structures are developed at the extremities of elongated cells, an oriented virions exocytosis through the Golgi apparatus in our cells cannot be totally ruled-out. Thirdly, under normal conditions, we also noticed at the periphery of the plaques that cell-cell junctions were visible between infected and non-infected cells with intercellular junctions enriched in actin cables reminiscent of AJs. Fourth and lastly, we demonstrated that MDV cell-to-cell spread was modulated in similar way by Rho/Rac manipulation than AJs stabilization and maintenance in non-infected cells. Indeed, under MDV cell-to-cell spread-compromising conditions, as upon treatment with Y-27632, the AJs were barely visible using an N-cadherin marker. In contrast, under conditions that are favorable for MDV spread, as upon treatment with LPA or NSC23766, the AJs were more readily detectable than in non-treated controls. This finding is in perfect line with the important role of Rho signaling in the formation and/or maintenance of AJs in mammalian myoblasts or fibroblasts [24], [59], [60]. For example, in C2C12 mouse myoblasts, Rac1 and RhoA were found to exert opposite effects, with RhoA promoting N-cadherin dependent cell-cell contacts. In addition, ROCK signaling and myosin II activation were found to stabilize N-cadherin at the cell-cell contact sites [59].

In conclusion, we showed for the first time that Rho/Rac pathways regulate MDV cell-to-cell spread. In particular, we found that Rho-ROCK pathway and actomyosin contraction is beneficial for MDV spread while Rac-PAK is detrimental. We also showed that the stabilization and maintenance of AJs, the only cell-cell junctions found to date in fibroblastic cells, are affected in a similar way by RhoGTPases manipulations than MDV cell-to-cell spread. Considering the cell-cell transmission of MDV, the importance of AJs or AJs proteins in alpha-herpesviruses entry or egress [10], [18], [81] and the rarity of cell extensions in our virus/cell system, we hypothesize that these junctions could be of special interest in MDV biology. In the future, our efforts will focus on deciphering a direct role of AJs in MDV cell-to-cell spread and to examine if MDV virions could be routed via this cell-cell contact.

## Materials and Methods

### Cells and viruses

Chicken embryo skin cells (CESCs) were prepared from 12 day-old, specific-free-pathogen Brown Leghorn (LD1) embryos exactly as previously described [82]. CESCs were cultivated at 41°C in William's E media (Lonza, Basel, Switzerland) supplemented with 2 mM of L-Glutamine (Lonza), 1% of fetal calf serum, 1.5% of chicken serum plus 5 mM of N,N′-Hexamethylene bisacetamide (HMBA) at confluency including during infection. At explantation, the monolayers contained mostly fibroblasts, a low percentage of myoblasts, for which the differentiation into myotubes was inhibited by HMBA [83], and rare keratinocytes. These cell types were identified on the basis of morphology and immunochemistry with a few cellular markers (N-Cadherin, Vimentin, integrin ß-1, Pax7 and Cytokeratin). Two BAC-derived MDV strains were used in this study: the highly virulent BAC RB-1B strain repaired for *UL13*, *UL44* (gC), *US2* and *US6* (gD) [58], [84], and the attenuated strain BAC20 [85]. One mutant virus generated from the BAC20 was used: the BAC20-EGFPVP22 (named herein BAC20-EGFP), which expresses the enhanced green fluorescent protein N-terminally fused to the viral VP22 tegument protein [34]. The sorted BAC20-EGFP-infected cells were obtained by flow cytometry on a MoFlo (DakoCytomation) with a purity above 95% as previously described [3].

### Pharmacological agents

Stock solutions were made in dimethyl sulfoxide (DMSO) for: cytochalasin D (CytD) (C8273, Sigma) at 20 mM, jasplakinolide (Jaspl) (J7473, Invitrogen) at 1 mM, IPA-3 (cat#506106, Calbiochem) at 20 mM, blebbistatin (Bleb) (B0560, Sigma) at 25 mM. The maximal final concentration of DMSO used in this study did not exceed 0.1%. Sphingosine-1-phosphate (S1P) (S9666, Sigma) at 1 mM, NSC23766 (National Cancer Institute) at 50 mM, L-alpha-lysophosphatidic acid, oleyl, sodium (LPA) (l7260, Sigma) at 1 mg/mL, Y-27632 (cat#688000, Calbiochem) at 5 mM and Fasudil, monohydrochloride salt (F-4660, LClabs) at 30 mM were dissolved in H_2_O as stock solutions.

### Purification of TAT-C3 transferase

The C3 transferase is a toxin from *Clostridium botulinum*, which was fused to a (His)_6_- and a HIV-1 TAT-tag at its N-terminus [86]. The expression in bacteria and purification of the TAT-C3 transferase has been described previously [87]. The purity of the protein was controlled by migration on a 12% SDS-PAGE followed by a Coomassie staining of the gel. The protein concentration was subsequently determined using the quick start Bradford Protein Assay (500-0203, Biorad). Before addition in CESCs culture media, TAT-C3 transferase was dialyzed overnight at 4°C against PBS (Slide-A-Lyzer® Dialysis Cassettes, Pierce), in order to eliminate the elution buffer containing imidazole at a concentration of 250 mM. Finally, the protein activity was controlled on non-confluent CESCs for its effect on actin organization.

### Immunofluorescence microscopy

Cells treated with drugs or purified TAT-C3 transferase were cultivated directly on 0.17 mm glass coverslips coated with 0.17% gelatin. Cells were fixed with 4% paraformaldehyde (PFA) for 30 min at room temperature, permeabilized and blocked with PBS, 0.1% Triton X-100, 0.5% BSA. F-actin was stained with AlexaFluor594-Phalloidin (1∶200) (A12381, Molecular Probes) and nuclei with Hoechst 33342 (1∶2,000) (H-3570, Molecular Probes). For AJs staining, cells were fixed with 4% PFA in the presence of 0.1% Triton, and the anti-N-cadherin mouse monoclonal antibody (1∶200) (GC-4, Sigma) was used and an AlexaFluor488 secondary antibody (A11001, Invitrogen). Cells were observed on an Axiovert 200 M inverted epi-fluorescence microscope equipped with a 40× PlanNeofluar oil/Dic objective or a 63× PlanApochromat oil/DIC, both with the ApoTome system (Zeiss). Images were captured with a CCD Axiocam MRm camera (Zeiss) using the Axiovision software (Zeiss).

### MDV cell-to-cell spread assays

The basic assay was as follows: approximately 10^6^ CESCs, plated in 3.5 cm diameter-wells (9.6 cm^2^), corresponding to a confluent monolayer, were infected by coculture with 3.2×10^3^ GFP-positive-sorted BAC20-EGFP-infected cells. After 6 hrs of infection, a pharmacological agent was added at different concentrations to the culture medium, and the cells were further incubated for 90 hrs. The drugs containing media were renewed at 48 hrs post-infection. For dialyzed TAT-C3 transferase protein, the assay was performed on 1.6 cm diameter plate (2 cm^2^) and the protein was renewed once at 30 hrs post-infection. In these cell culture formats, for unkown reasons, plaques sizes were reproducibly smaller than in larger plates (>9.6 cm^2^). After the 4-day incubation period, cells were fixed by 4% PFA. Plaques were detected with a Fluar ×5 objective or a 5× EC plan-neofluar/ph1 mounted on an inversed Axiovert 200 M inverted epi-fluorescence microscope (Zeiss). Plaques size measurements were performed on images captured from 50 plaques through a CCD Axiocam MRm camera using the Axiovision software (Zeiss) as previously described [34]. Plaque areas were measured with Axiovision software with the outline measure tool, by manually drawing the outline of each plaque. When the outline was closed, the surface area was automatically calculated in µm^2^. Plaque sizes after drug treatment were averaged over 50 plaques using Prism 5.0 software (GraphPad Prism software version 5.01), and mean plaque sizes for each experiment was analyzed using the Kruskal-Wallis test. When significant differences were detected, a Dunn test was used to compare each condition with the control. Plaque size measurements after each drug treatment was performed in two to three independent experiments. As the plaques size may varies for a same virus according to the cell density, an internal control was introduced in each independent experiment and the various conditions compared to it.

When the BAC20 virus was used, the duration of spread was reduced to 3 days as this virus grows faster and leads to higher plaques sizes on cell monolayer than BAC20-EGFP and the BAC RB-1B. When a cell monolayer was infected with a non-fluorescent MDV as the BAC20 or the BAC RB-1B, infection plaques were revealed by staining with a cocktail of three mouse monoclonal antibodies (F19 anti-VP5, K11 anti-gB, and E21 anti-ICP4; each diluted at 1∶1,000) followed by a goat anti-mouse AlexaFluor® 488 (Invitrogen, Carlsbad, CA, USA) as described [88]. Next, plaques sizes were measured on the basis of the green fluorescence as described above.

To study differences in plaques shape between several conditions, the following analysis was performed: after having outlined the plaque areas, a first segment between the two most distant points of the outline was measured with Axiovision software and defined as length. A second segment, perpendicular to the first and crossing it in its mid was outlined and measured, and defined as width. For 30 plaques, the ratio between plaque length on plaque width was next calculated and plotted on a graph. Statistical analyses were performed as described above.

### Cell viability assay

Uninfected CESCs were cultivated in 24-well plates in the presence of the highest concentration of drugs or TAT-C3 transferase protein using the same conditions as described for the MDV cell-to-cell spread assay but without MDV infection. After a 4-day treatment, the AlamarBlue viability reagent (DAL1025, Invitrogen) was added into the cell culture media, according to the manufacturer's recommendations. After 3 hrs, the cell viability was determined by monitoring the substrate fluorescence with a Spectramax Gemini EM spectrofluorimeter and with the SoftMaxPro software (Molecular Devices). Results were reported as the ratio between the fluorescence units measured for the treated cells and non-treated cells. For each drug, this assay was performed at least in two independent experiments and in triplicate. For each condition, the SEM was calculated. We set the threshold of cell viability acceptable for our cell-to-cell spread assay at 75% of the mock-treated cells. This threshold corresponds to the cell viability measured for the treatment with the NSC23766 at 40 µM, and for which we observed an increase of cell-to-cell MDV spread.

### Gel electrophoresis and immunoblotting to detect the phosphorylation of myosin light chain (MLC)

Cells cultivated in 6 cm diameter-dishes, treated or not with Y-27632 or Fasudil, were rinsed once in PBS and directly lyzed in electrophoresis sample buffer 2× containing 120 mM Tris-HCl (pH 6.8), 20% glycerol (vol/vol), 4% SDS, 2.5% ß-mercaptoethanol, 2 mM EGTA, 0.01% Bromophenol blue. Lysates were boiled for 3 minutes, separated on 12% SDS-PAGE, transferred onto a nitrocellulose membrane (cat#741290 Porablot NCL, Macherey-Nagel) through a semi-dry system and blotted as follow. The membrane was incubated either with a mouse monoclonal anti-Glyceraldehyde-3-phosphate dehydrogenase (GAPDH) antibody (1∶500) (cat#MAB374, Chemicon-Millipore) or a rabbit anti-Phospho-Myosin Light Chain (Thr18/Ser19) (1∶1000) (cat#3674, Cell Signaling) overnight at 4°C and an alkaline phosphatase-conjugated secondary antibody. The alkaline phosphatase was detected with a solution made of nitro Blue tetrazolium chloride (NBT) and 5-bromo-4-chloro-3-indolyl phosphate (BCIP) (Zymed).
